# Antioxidant Capacity of Selected Plant Extracts and Their Essential Oils 

**DOI:** 10.3390/antiox2010011

**Published:** 2013-01-04

**Authors:** Charalampos Proestos, Konstantina Lytoudi, Olga Konstantina Mavromelanidou, Panagiotis Zoumpoulakis, Vassileia J. Sinanoglou

**Affiliations:** 1 Laboratory of Food Chemistry, Department of Chemistry, National and Kapodistrian University of Athens, Athens 15771, Greece; E-Mails: konstantinalyt@hotmail.com (K.L.); o_mav@hotmail.com (O.K.M.); 2 Institute of Biology, Medicinal Chemistry & Biotechnology, National Hellenic Research Foundation, 48, Vas. Constantinou Ave., Athens 11635, Greece; E-Mail: pzoump@eie.gr; 3 Laboratory of Food Analysis, Department of Food Technology, Technological Educational Institution of Athens, Egaleo 12210, Greece; E-Mail: v_sinanoglou@teiath.gr

**Keywords:** plant extracts, essential oils, HPLC-UV/vis, antioxidant capacity, DPPH, ABTS, Rancimat test

## Abstract

The main objective of this study was the screening of some selected aromatic plants very popular in Greece, with respect to their total phenolic content, antioxidant capacity, reducing activity, and oxidative stability. All plants were extracted with the conventional method, reflux with methanol. The essential oils of the plants were also analyzed for their antioxidant properties. The total phenolic content was determined by the Folin-Ciocalteu method using gallic acid as the standard, while the phenolic substances were identified and quantified by High Performance Liquid Chromatography (HPLC) coupled with a multi-wavelength ultraviolet-visible (UV-vis) detector. The antioxidant capacity of the plant extracts was measured by their ability to scavenge free radicals such as (a) DPPH (2,2-diphenyl-1-picrylhydrazyl) and, (b) ABTS (2,2′-azinobis-(3-ethylbenzothiaziline-6-sulfonate). The Folin-Ciocalteu method proved the existence of antioxidants in the aromatic plant extracts. Taking into account the results of the DPPH and ABTS methods, the free radical scavenging capacity was confirmed. Eventually, all plants exhibited low but noticeable protection levels against lipid oxidation, as determined by the Rancimat test.

## 1. Introduction

In an effort to minimize the undesirable effects of synthetic food preservatives in human health, food industries and scientists have recently turned their interest to new preservatives. Aromatic plants are well known for their antioxidant and antimicrobial properties that prevent food degradation and alteration [1], as they are rich in phenolic substances, usually referred to as polyphenols, which are ubiquitous components of plants and herbs. Halliwell and Gutteridge [2] defined antioxidants as compounds that—when present in low concentration in relation to the oxidant—prevent or delay the oxidation of the substrate. Their importance in the safeguarding of health, and the protection from coronary heart disease and cancer, has recently been established, thus constituting them as functional food preservatives.

Polyphenols are antioxidants with redox properties, which allow them to act as reducing agents, hydrogen donators, and singlet oxygen quenchers. Some show metal chelation properties [3,4]. In addition, some have antimicrobial activity [5]. A great number of aromatic plants have been reported as having anti-inflammatory, antiallergic, antimutagenic, antiviral, antithrombotic, and vasodilatory actions [6]. High performance liquid chromatography (HPLC) is a high resolution chromatographic technique that is very common for the simultaneous separation and quantification of phenolic substances without any preliminary treatment. In this study, the identification of each compound was based on a combination of retention time and spectral matching.

The main objectives of the study are the characterization and quantification of the phenolic fraction of plant extracts and essential oils; the evaluation of the antioxidant activity of extracts and essential oils by using specific assays; and, a comparison of the antioxidant activity results obtained from the different assays employed.

## 2. Experimental Section

### 2.1. Plant Materials and Reagents

The part of the plants examined and the drying method used are presented in Table 1. Dried samples were purchased from the local market or collected from different sites in Greece. All samples were analyzed within three months of collection. Gallic acid, gentisic acid, *p*-coumaric acid, vanillic acid, ferulic acid, syringic acid, (+)-catechin, quercetin, apigenin, naringenin, myricetin, were purchased from Sigma-Aldrich (Steinheim, Germany). Luteolin was from Roth (Karlsruhe, Germany). Caffeic acid was from Merck (Darmstadt, Germany). (−)-Epicatechin was from Fluka AG (Buchs, Switzerland). Rutin was from Alexis Biochemicals (Lausen, Switzerland). Ascorbic acid, *p*-hydroxybenzoic acid, and BHT (butylated hydroxytoluene) were a kind donation from the National Agricultural Research Foundation (N.AG.RE.F, Athens, Greece). Quantification was done via calibration with standards (external standard method). All standards were prepared as stock solutions in methanol. Methanol and sodium carbonate (Na_2_CO_3_) were purchased from Carlo Erba-SDS. Working standards were made by diluting stock solutions in 62.5% aqueous methanol containing BHT 1 g/L, and 6mol/L HCL to yield concentrations ranging between 0.5 and 25 mg/L. Stock/working solutions of the standards were stored in darkness at −180 °C. All solvents and reagents from various suppliers were of the highest purity needed for each application. The Folin-Ciocalteu reagent was purchased from Merck (Darmstadt, Germany). 2,2-diphenyl-1-picrylhydrazyl (DPPH) was obtained from Sigma-Aldrich (Steinheim, Germany). The ABTS reagent (2,2′-azinobis-(3-ethylbenzothiaziline-6-sulfonate) was from MP Biomedicals, (Eschwege, Germany).

### 2.2. Extraction Procedure

The extraction method used for dried samples had as follows: 50 mL of methanol were added to 1 g of dried sample. The extraction mixture was refluxed in a water bath at 70 °C for 1 h. The mixture was then filtered and placed in a test tube in the fridge. 

The essential oils were prepared by hydrodistillation, 30 g of plant materials were added in 300 mL water, for 3 h using a Clevenger-type apparatus. The supernatant was separated by decantation, dried over anhydrous Na_2_CO_3_ and kept in sterile flasks.

To prevent oxidation, all steps were carried out in dark with the flasks covered with aluminum foil. Moreover, it is essential that the waste (due to evaporation) of the solvent was kept at the lowest minimum.

The extraction method for the HPLC analysis had as follows: 40 mL of 60% aqueous methanol containing BHT (1 g/L) to prevent oxidation of phenolics extractedwas added to 0.5 g of dried sample. Then 10 mL of 6 mol/L HCl were added (separate aglycones from glycosides). The mixture was stirred carefully. In each sample nitrogen was bubbled for *ca.* 40–60 s. The extraction mixture was then sonicated for 15 min and refluxed in a water bath at 90 °C for 2 h. The mixture was then filtered and made up to 100 mL with methanol furthermore filtered quickly through a 0.45 μm membrane filter (Millex-HV) and injected to HPLC.

### 2.3. Determination of Total Phenolics

The total phenol content (TPC) was measured using the Folin-Ciocalteu assay [3]. A volume of 0.2 mL of the extract was introduced into test tubes followed by 0.5 mL Folin-Ciocalteu’s reagent (diluted 10 times with water). The solution was then kept at dark for 5 min and then 1 mL sodium carbonate (7.5% w/v) was added. The tubes were covered with parafilm and kept again in the dark for 1 h. Absorption at 765 nm was measured with a spectrophotometer UV-vis (Jasco V-530) and compared to a gallic acid calibration curve. The results were expressed as mg gallic acid/g dried sample. Each assay was carried out in triplicate. The determination of the total phenol content of the essential oils was done as follows: a volume of 3 mL of the methanol solution of each essential oil was introduced into test tubes followed by 1 mL Folin-Ciocalteu’s reagent (diluted 10 times with water) and 1 mL sodium carbonate (7.5% w/v).

### 2.4. Determination of Antioxidant Activity Using the 2,2-Diphenyl-1-picrylhydrazyl (DPPH) Radical Scavenging Method

The antioxidant activity was measured in terms of hydrogen donating or radical scavenging ability using the stable radical DPPH. Experiments were carried out according to the method of Blois [7] with a slight modification [8]. The reduction of the radical is followed by a decrease in the absorbance at 517 nm. A volume of 2 mL of a methanolic or aqueous methanolic stock solution of the extracts was put into test tubes and 2 mL of 1 mM DPPH solution was added. The tubes were covered with parafilm and kept again in the dark for 1 h. Absorbance at 517 nm was measured with a spectrophotometer UV-vis (Jasco V-530) and compared to an ascorbic acid calibration curve. The results were expressed as mg ascorbic acid/g dried sample. Each assay was carried out in triplicate. The percentage inhibition of the DPPH radical was calculated using the following formula:

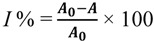
(1)
where *I* = DPPH inhibition (%), *A_0_* = absorbance of control sample (*t* = 0 h) and *A* = absorbance of a tested sample at the end of the reaction (*t *= 1 h).

The amount of sample necessary to decrease the absorbance of DPPH by 50% (IC_50_) was calculated graphically for the methanolic solutions of the essential oils of rosemary, thyme, oregano, and sage in six different concentrations. 

### 2.5. Determination of Antioxidant Activity Using the ABTS Free Radical Scavenging Method

The ABTS radical cation decolorization method is based on the reduction of ABTS^•+^ radicals by antioxidants of the plant extracts tested. The reaction’s mechanism involves the electron-donating ability and results in the decolorization of the radical. ABTS was first dissolved in deionized water to a 7-mM concentration. Then, the solution of K_2_S_2_O_8_ was prepared at a concentration of 2.45 mM. The two solutions were mixed with a ratio of 1:1 and kept in the dark for 24–48 h. The ABTS solution was then diluted in aqueous methanol with a ratio of 1:25. A volume of 20 μL (diluted 1:10) of aqueous methanolic plant extract was added to 2 mL of ABTS^•+^ solution, and the mixture was kept at a standard temperature of 30 °C. The absorbance was measured at 734 nm in 0, 5, and 10 min after initial mixing. All solutions were used on the day of preparation, and all determinations were carried out in triplicate. The results were expressed as mg ascorbic acid/g dried sample. The percentage of inhibition of ABTS^•+^ was calculated using the following formula:

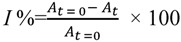
(2)
where *I* = DPPH inhibition (%), *A_t=__0_* = absorbance of control sample (*t* = 0 h) and *A_t_* = absorbance of a tested sample in 5 or 10 min. Antioxidant capacity was expressed in mg ascorbic acid/g dried sample. 

### 2.6. Rancimat Test

Samples of sunflower oil (3.5 g) containing 0.2% w/w extract were subjected to oxidation at 110 °C (air flow 20 L/h) [9]. The standard compounds (0.2% addition) were also examined. The rate of auto-oxidation of sunflower oil was estimated according to the increase in the induction period (IP). In the case of an increase in the IP, the extract displayed antioxidant ability, whereas a decrease in the IP indicated oxidative ability. The protection factors and the antioxidant activity of the samples were calculated according to the following formulas [10]:

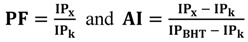
(3)
where IP_x_ = induction period of the sample with additive, IP_k_ = induction period of sample without additive, IP_BHT_ = induction period of sample with added synthetic antioxidant BHT.

### 2.7. HPLC Analysis

The analytical HPLC system employed consisted of a JASCO high performance liquid chromatograph coupled with a UV-vis multi-wavelength detector (MD-910 JASCO). The analytical data were evaluated using a JASCO data processing system (DP-L910/V). The separation was achieved on a Waters Spherisorb^®^ 5μm ODS2 4.6 × 250 mm column at ambient temperature. The mobile phase consisted of water with 1% glacial acetic acid (solvent A), water with 6% glacial acetic acid (solvent B), and water-acetonitrile (65:30, v/v) with 5% glacial acetic acid (solvent C). The gradient used was: 100% A 0–10 min, 100% B 10–30 min, 90% B/10% C 30–50 min, 80% B/20% C 50–60 min, 70% B/30% C 60–70 min, 100% C 70–105 min, 100% A 105–110; *post* time 10 min before next injection [11]. The flow rate was 0.5 mL/min and the injection volume was 20 μL. The monitoring wavelength was 280 nm for the phenolic acids and 320 and 370 nm (flavones, flavonoles). The identification of each compound was based on a combination of retention time and spectral matching.

## 3. Results and Discussion

### 3.1. Determination of Total Phenolics

The results of the determination of total phenolics are demonstrated in Table 1. *Nepeta melissifolia*, *Phlomis lanata *and *Origanum vulgare* demonstrated the highest total phenol content with more than 15.0 mg gallic acid/g dried sample*. Geranium purpureum*, *Matricaria chamomilla* and *Lavandula vera* seemed to have the lowest antioxidant capacity with less than 7 mg gallic acid/g dried sample. The results of this study were comparable to other similar ones [12,13,14]. Similar amounts in plant phenolics have also been reported from herbs and medicinal plants collected in Finland [3].

The methanolic solution of the essential oil of thyme showed the highest antioxidant capacity with 18 mg gallic acid/g dried sample (Table 2). The essential oil of rosemary was found to contain a relatively low concentration of phenols with only half that present in the essential oil of thyme. Viuda-Martos [14] demonstrated similar results. These results indicated that the phenolic compounds had a major contribution to the antioxidant capacity of herbs.

**Table 1:** Total phenolics in plant extracts.

Family Species	Collection sites	Part examined	Drying method ^a^	Total phenolics ^b^ (mg gallic acid/g ds)
*Origanum dictamnus *(A)	Crete	Leaves	Air	8.2 ±0.3
*Eucalyptus globulus *(B)	Attiki	Leaves	Air	10.5 ± 0.3
*Sideritis cretica *(C)	Crete	Leaves	F/v	8.6 ± 0.2
*Origanum vulgare *(D)	Euboea	Leaves	F/v	19.5 ± 0.2
*Phlomis cretica *(E)	Crete	Leaves	F/v	16.2 ± 0.1
*Phlomis lanata *(F)	Crete	Leaves	F/v	21.4 ± 0.3
*Nepeta melissifolia *(G)	Crete	Leaves	F/v	31.6 ± 0.4
*Mentha pulegium *(H)	Crete	Leaves	F/v	13.4 ± 0.2
*Thymus vulgaris *L. (I)	Attiki	Leaves	Air	8.0± 0.1
*Satureja thymbra *(J)	Attiki	Leaves	Air	9.2± 0.1
*Lavandula vera *(K)	Attiki	Leaves	Air	4.9± 0.1
*Rosmarinus officinalis *(L)	Attiki	Leaves	Air	8.5± 0.1
*Lippia triphylla *(M)	Attiki	Leaves	Air	7.7± 0.1
*Matricaria chamomilla *(N)	Attiki	Leaves	Air	6.1± 0.1
*Mellisa officinalis *L.(O)	Attiki	Leaves	Air	15.1 ± 0.1
*Salvia officinalis *(P)	Attiki	Leaves	Air	15.6 ± 0.1
*Geranium purpureum *(Q)	Attiki	Leaves	Air	4.0 ± 0.1

^a^ Air = air drying; F/v = Freeze vacuum, *i.e.*, lyophilization; ^b^ Mean of triplicate assays; ds = dry sample.

**Table 2:** Total phenolics in essential oils.

Family Species	Total phenolics ^a^ (mg gallic acid/g ds)
*Thymus vulgaris* L.	18.0 ± 0.9
*Salvia officinalis*	12.1 ± 0.1
*Rosmarinus officinalis*	9.2 ± 0.1
*Origanum vulgare*	±0.2

^a^ Mean of triplicate assays; ds = dry sample.

### 3.2. Determination of Antioxidant Activity Using the 2,2-Diphenyl-1-picrylhydrazyl (DPPH) Radical Scavenging Method

**Figure 1:**
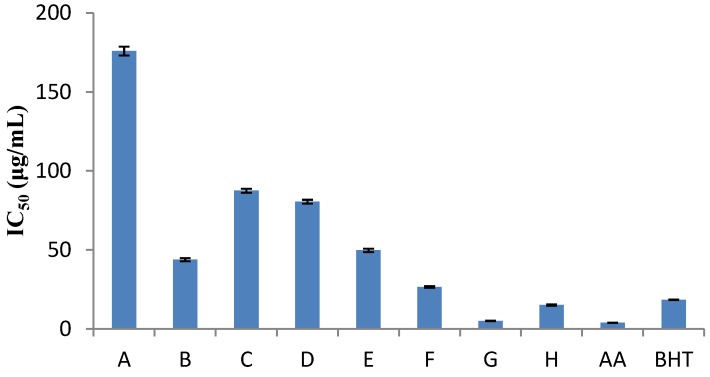
Free radical scavenging activities, where: (**A**) *Origanum dictamnus*; (**B**) *Eucalyptus globulus*; (**C**) *Sideritis cretica*; (**D**) *Origanum vulgare*; (**E**) *Phlomis cretica*; (**F**) *Phlomis lanata*; (**G**) *Nepeta melissifolia*; (**H**) *Mentha pulegium*, and **AA** and **BHT** stand for ascorbic acid and butylated hydroxytoluene, respectively.

**Figure 2:**
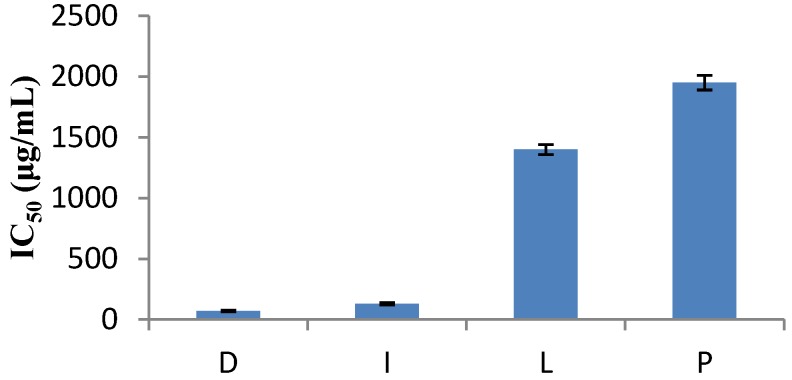
Free radical scavenging activities of some essential oils.

The DPPH method was evidently introduced nearly 50 years ago by Blois [7] and it is widely used to test the ability of compounds to act as free radical scavengers or hydrogen donors, and to evaluate antioxidant capacity. The parameter IC_50_ (“efficient concentration” value), is used for the interpretation of the results from the DPPH method and is defined as the concentration of substrate that causes 50% loss of the DPPH activity (color). Some plant extracts and essential oils were examined in relation to their IC_50_ value, while others were tested for their antioxidant capacity. IC_50_ values of *Nepeta melissifolia*, *Mentha pulegium* and *Phlomis lanata* (5.1 ± 0.4, 13.5 ± 0.5, 23.9 ± 0.4 μg/mL) were found to be similar to BHT and ascorbic acid (18.5 ± 0.4, 3.9 ± 0.3 μg/mL). The IC_50_ values of the examined plant extracts and essential oils are presented in Figure 1 and Figure 2, respectively.

As the IC_50 _concentration and the antioxidant capacity have inversely proportional values, *Salvia officinalis* was established to have the lowest antioxidant capacity while *Origanum vulgare* was found to be the richest of all. Similar studies in plant extracts [15] exhibited higher hydrogen-donating capacity.

For the majority of the plants, as shown in Table 3, the DPPH method showed low antioxidant capacity. Only *Sideritis cretica * and *Geranium purpureum* were found to be the richest in antioxidants, approaching 1 mg ascorbic acid/g dried sample.

**Table 3:** Antioxidant capacity of plant extracts.

Family species	Antioxidant Capacity (mg ascorbic acid/g dried sample)
*Thymus vulgaris *L.	0.6 ± 0.3
*Lavandula vera*	0.6 ± 0.4
*Rosmarinus officinalis*	0.5 ± 0.1
*Origanum dictamnus*	0.2 ± 0.2
*Sideritis cretica*	0.8 ± 0.1
*Salvia officinalis*	0.4 ± 0. 1
*Origanum vulgare*	0.5 ± 0.1
*Geranium purpureum*	±0.2

### 3.3. Determination of Antioxidant Activity Using the ABTS Free Radical Scavenging Method

The results of the determination of antioxidant activity are demonstrated in Table 4. *Origanum vulgare*, * Origanum dictamnus* and *Thymus vulgaris* L. indicated the highest % inhibition for 0–5 min. In most cases as time elapsed, the % inhibition decreased due to the fact that the antioxidants of the aromatic plants scavenge the cation radical ABTS^•+^. The values found in the present study were also comparable to similar research work [16,17].

**Table 4:** % inhibition measured by the ABTS method for 0–5 min and 5–10 min.

Family species	% Inhibition (0–5 min)	% Inhibition (5–10 min)
*Thymus vulgaris* L.	21.1 ± 0.,2	10.9 ± 0.3
*Satureja thymbra*	19.2 ± 0.3	20.42 ± 0.4
*Lavandula vera*	18.2 ± 0.1	15.7 ± 0.3
*Rosmarinus officinalis*	15.6 ± 0.2	9.8 ± 0.5
*Lippia triphylla*	14.3 ± 0.3	7.2 ± 0.2
*Matricaria chamomilla*	12.4 ± 0.1	11.9 ± 0.5
*Origanum dictamnus*	22.7 ± 0.1	24.2 ± 0.3
*Melissa officinalis *L.	11.8 ± 0.2	38.6 ± 0.2
*Sideritis *spp.	12.2 ± 0.1	8.7 ± 0.4
*Salvia officinalis*	13.2 ± 0.3	9.6 ± 0.4
*Origanum vulgare*	25.5 ± 0.3	14.7 ± 0.3
*Geranium purpureum*	10.8 ± 0.2	12.5 ± 0.1

### 3.4. Rancimat Test—Method of Assessment of Oil Oxidation and Stability

The outcome of the Rancimat test as shown in Table 5 supports the hypothesis that aromatic plants are good sources of natural antioxidants such as phenolic compounds. When working accurately, this method offers an efficient, simple and automated assay. When ground material was added to sunflower oil, protection factors were slightly higher compared to the addition of methanol extracts. Similar PF values for ethanol and acetone extracts of plants of Greek origin have been reported [9]. 

**Table 5:** Antioxidant capacity of plant extracts in PF values.

Family Species	PF ^α^ (ground material)	PF ^α^ (methanol extracts)
*Thymus vulgaris* L.	ND ^b^	1.7 ± 0.05
*Rosmarinus officinalis*	ND	2.0 ± 0.06
*Origanum vulgare*	1.8 ± 0.09	1.9 ± 0.06
*Origanum dictamnus*	1.3 ± 0.08	1.2 ± 0.09
*Eucalyptus globulus*	1.5 ± 0.07	1.4 ± 0.08
*Sideritis cretica*	1 ± 0.06	1.1 ± 0.07
*Phlomis cretica*	2.1 ± 0.07	1.9 ± 0.07
*Phlomis lanata*	2.4 ± 0.09	2.1 ± 0.06
*Nepeta melissifolia*	3.1 ± 0.06	2.9 ± 0.09
*Mentha pulegium*	1.9 ± 0.05	1.7 ± 0.07

^a^ PF = Protection Factor, ^b^ ND= Not detected.

Based on the table above, most aromatic plants indicated low protection against oil oxidation. Yet, the ground material of *Nepeta melissifolia* demonstrated very high protection while *Sideritis cretica* was found to have pro-oxidative action. Similar values were also established by Kintzios *et al.* [18] for the aromatic plants of Labiatae family. Aruoma *et al*. [19] found that *Rosmarinus officinalis* had medium protection (PF: 2.36) against oil oxidation in contrast with *Thymus vulgaris* L. and *Origanum vulgare* that indicated low protection.

### 3.5. HPLC Analysis

The present method is simple, easy to use, and effective enough for the identification and quantification of major phenolic compounds in aromatic plants. This has been reported by other authors, who have used the same method of extraction and analysis of major flavonoid aglycones [1,20,21]. Generally, reversed phase HPLC with C_18_ columns is the most popular technique for the analysis of polyphenols in different foods, despite the fact that the separation of procyanidins is not satisfactory with these phases [21]. Spherisorb C_18_ stationary phase, which was used in this study to separate phenolic acids and flavonoids in aromatic plants, produced quite good results. After extraction and acid hydrolysis, the content of phenolic substances was determined. After extraction and acid hydrolysis the content of phenolic substances was determined. Quantification was done via a calibration with standards (external standard method). The amount of phenolic acids detected in the analyzed samples is shown in Table 6. Additionally, the content of flavonoids identified in the same plant extracts is shown in Table 7. Results are expressed in mg/ 100g dry sample. (+)-Catechin, quercetin and rutin were the most abundant flavonoids. Quercetin has been reported to be present in a large number of aromatic plants [1]. Rutin (quercetin 3-o-rhamnose glycoside) was present along with 22 other flavones, flavonoles and their glycosides in tea leaves [22]. Rutin in some cases can be hydrolyzed to quercetin (aglycone). This could have happened in the examined samples. Caffeic acid was detected in all the examined plant extracts. Ferulic acid was also detected in all the methanolic extracts, except from *P. lanata*, in rather high concentration. Phenolic acids are found in nature as esters and rarely as glycosides or in free form [23]. General comments have been published [12,24,25]. Thus, hydrolysis was needed for the identification and quantitative determination of phenolic acids. The data presented in Table 6 and Table 7 are considered as indicative of phenolic content of these aromatic plants. Papers about most of the examined plant extracts are very scarce in the literature. Among others reasons, time of harvest and storing conditions are considered responsible for the observed variations in the phenolic contents.

**Table 6:** Content of phenolic acids in the examined plant extracts.

Family species	gallic acid	gentisic acid	caffeic acid		*p*-coumaric acid	vanillic acid	syringic acid	ferulic acid	*p*-hydroxybenzoic acid
*Origanum dictamnus *(A)	4.9 ± 0.03	ND	13.5 ± 0.02		13.9 ± 0.04	18.5 ± 0.02	ND	16.9 ± 0.04	ND
*Eucalyptus globulus* (B)	ND	ND	8.1 ± 0.01		6.6 ± 0.02	ND	ND	12.3 ± 0.03	ND
*Sideritis cretica* (C)	1.1 ± 0.02	ND	3.3 ± 0.02	ND	ND	ND	6.8 ± 0.02	2.5 ± 0.01
*Origanum vulgare* (D)	ND	ND	6.4 ± 0.02	ND	ND	ND	10.4 ± 0.03	ND
*Phlomis cretica* (E)	ND	ND	2.2 ± 0.01	ND	ND	ND	5.1 ± 0.02	ND
*Phlomis lanata* (F)	14 ± 0.02	3.2 ± 0.03	20 ± 0.03	4.1 ± 0.02	2 ± 0.02	1.1 ± 0.02	ND	1.5 ± 0.01
*Nepeta melissifolia* (G)	20 ± 0.02	4.3 ± 0.03	26 ± 0.03	5.2 ± 0.02	2.7 ± 0.02	2.6 ± 0.02	22.4 ± 0.03	5.4 ± 0.01
*Mentha pulegium* (H)	ND	ND	13.5 ± 0.02	ND	13.5 ± 0.02	ND	13.5 ± 0.02	ND

Each value is the mean (mg/100 g dry sample) of two replications standard deviation; ND = Not detected.

**Table 7:** Flavonoid content in the examined plant extracts.

Family species	quercetin	apigenin	luteolin	naringenin	myricetin	rutin	(+)-catechin hydrated	(−)-epicatechin
*Origanum dictamnus*	52 ± 0.09	ND	ND	ND	ND	ND	1.9 ± 0.01	ND
*Eucalyptus globulus*	ND	ND	ND	ND	ND	10 ± 0.03	ND	ND
*Sideritis cretica*	ND	ND	ND	ND	ND	ND	6.9 ± 0.02	2.8 ± 0.01
*Origanum vulgare*	7.3 ± 0.02	ND	ND	ND	ND	2.3 ± 0.01	2.5 ± 0.01	ND
*Phlomis cretica*	1.2 ± 0.01	ND	ND	ND	ND	ND	1.5 ± 0.01	2.6 ± 0.01
*Phlomis lanata*	2.2 ± 0.01	ND	ND	ND	ND	4.5 ± 0.01	ND	ND
*Nepeta melissifolia*	11.2 ± 0.02	5.6 ± 0.02	ND	ND	1.8 ± 0.01	2.4 ± 0.01	5.5 ± 0.02	ND
*Mentha pulegium*	ND	6.9 ± 0.02	ND	4.7 ± 0.02	ND	ND	3.5 ± 0.01	ND

## 4. Conclusions

In the present study, some Greek aromatic plants were tested with respect to their total phenolic content, antioxidant capacity, and oxidative stability. Extractions were performed using the conventional method reflux and methanol as solvent. Four aromatic plants were tested also for the antioxidant capacity of their essential oil. The existence of phenolic compounds in the aromatic plants was confirmed by the Folin-Ciocalteu method. The antioxidant capacity was measured by the free radical scavenging methods DPPH and ABTS and was proven to be high. The methanolic solutions of the essential oils showed high antioxidant capacity as proved by their low IC_50_ concentration. All aromatic plants exhibited low reducing power, which are resultscomparable to similar studies. Moreover, according to the protect factor scale, the ground material indicated higher protection against oil oxidation and stability than the methanol extracts. Finally, the results in this study indicate that the examined aromatic plants contain certain amounts of polyphenols and flavonoids, proving them to be perfect sources of antioxidants.
